# Macrophage-Rich Peritoneal Compartments and CCR2-Dependent Hematopoietic Recruitment Are Differentially Associated with Adhesion Formation After Abdominal Surgery

**DOI:** 10.3390/biomedicines14092014

**Published:** 2026-09-08

**Authors:** Anna Woestemeier, Mariola Lysson, Lara Braun, Azin Jafari, Philipp Lingohr, Sven Wehner, Jörg C. Kalff, Gun-Soo Hong

**Affiliations:** Department for General, Visceral, Vascular Surgery, University Hospital of Bonn, 53127 Bonn, Germany

**Keywords:** postoperative adhesion formation, macrophages, CCR2, monocytes, ischemic button, peritoneal inflammation

## Abstract

**Background:** Postoperative peritoneal adhesions arise from a dysregulated wound-healing response in which macrophages may have context-dependent effects. We investigated the contribution of macrophage-rich peritoneal and mesenteric compartments, the origin of macrophage-like cells in ischemic lesions, and the association between CCR2-dependent recruitment and postoperative inflammatory and reparative gene expression. **Methods:** Using a murine ischemic-button model, we assessed the effects of clodronate liposome treatment, bone marrow chimerism, and global CCR2 deficiency. Adhesion formation, F4/80+ cell accumulation, donor-marker expression, and selected inflammatory and wound-healing-associated transcripts were analyzed at predefined postoperative time points. **Results:** Clodronate liposome treatment was associated with reduced adhesion formation and substantial depletion of F4/80+ cells in peritoneal lavage and mesenteric tissue. However, F4/80+ cell numbers within ischemic buttons at postoperative day 3 were not significantly reduced, indicating that the depletion experiment does not establish selective depletion of all lesional macrophages. Bone marrow chimera experiments identified donor-marker-positive, F4/80+ cells within ischemic buttons, supporting recruitment of hematopoietic cells with a macrophage-like phenotype. CCR2 deficiency reduced F4/80+ cell accumulation in ischemic buttons and was associated with increased adhesion scores and altered expression of inflammatory and wound-healing-associated genes. These findings identify differential associations of clodronate-sensitive macrophage-rich compartments and CCR2-dependent hematopoietic recruitment with postoperative adhesion formation. **Conclusions:** Depletion of macrophage-rich peritoneal and mesenteric compartments was associated with reduced adhesion formation, whereas global CCR2 deficiency was associated with fewer lesional F4/80+ cells and greater adhesion severity. Because clodronate depletion, F4/80 staining, bone marrow chimerism, and global CCR2 deficiency do not provide cell-specific or fate-mapped resolution, these data do not establish distinct resident versus infiltrating macrophage functions or a reparative phenotype of CCR2-dependent cells. Cell-specific and temporally resolved validation is required to define the contributions and temporal relationships of individual macrophage subsets.

## 1. Introduction

Postoperative peritoneal adhesions remain a frequent and clinically significant complication of abdominal and pelvic surgery, affecting the majority of patients to varying degrees after operative intervention. Adhesions are associated with substantial morbidity, including chronic abdominal or pelvic pain, small bowel obstruction, female infertility, difficulties during reoperative surgery, and increased risk of inadvertent enterotomy. They also impose a considerable socioeconomic burden through hospital readmissions, prolonged healthcare utilization, and the need for additional surgical procedures. Their formation reflects an exaggerated and dysregulated wound-healing response in which immune cells, fibrin deposition, mesothelial repair, and tissue remodeling interact closely. To develop better preventive treatments for postoperative abdominal adhesions, the cellular and molecular basis of adhesion formation must be better understood [1,2,3,4].

Macrophages have emerged as important regulators of postoperative tissue responses, but prior work suggests that their effects are context-dependent. Resident peritoneal macrophages are long-lived, tissue-adapted cells that normally survey the peritoneal cavity, clear debris, and rapidly respond to sterile injury. Circulating monocytes can also be recruited from the blood after tissue damage, in part through CCR2-dependent pathways, and may differentiate locally into monocyte-derived macrophages whose phenotypes are shaped by the tissue environment [5,6,7].

Thus, in pathological situations, macrophages are a heterogeneous population. Recent single-cell studies further demonstrate that peritoneal macrophages comprise multiple transcriptionally and functionally distinct states, including resident, transitory, monocyte-derived, scar-associated, and pro-resolving populations. These states are influenced by ontogeny, tissue localization, and the inflammatory microenvironment, supporting a model that extends beyond the conventional M1/M2 classification; however, phenotypic overlap and state transitions make binary resident-versus-infiltrating assignments difficult without lineage-resolved approaches [8]. In the peritoneal cavity, resident macrophages may act as early responders that organize fibrin containment and mesothelial repair, yet under some conditions, they also contribute to adhesion formation by promoting cell aggregation and scar-like remodeling. By contrast, infiltrating macrophages derived from circulating monocytes may either amplify injury or support resolution, depending on the inflammatory milieu and the phase of healing. This heterogeneity is important because the postoperative lesion is shaped not only by the presence of macrophages, but by the balance between resident and circulating cell populations. Recent single-cell analyses further show that resident large peritoneal macrophages, recently recruited monocyte-derived macrophages, and transitional macrophage states exhibit different transcriptional programs and may have divergent effects on tissue remodeling and resolution [3,8,9,10,11].

Macrophages can express overlapping inflammatory and wound-healing-associated transcriptional programs. Although M1/M2 terminology remains common, it does not define discrete in vivo populations. In the present study, IL1B, TNF, and IL6 were treated as inflammatory readouts, whereas ARG1, MRC1, and IL10 were interpreted as wound-healing-associated transcripts rather than as evidence of a cell-specific M1 or M2 phenotype [12,13,14].

CCR2 is a key regulator of monocyte egress from the bone marrow and recruitment to inflamed tissues, including the peritoneal cavity, making CCR2 deficiency useful for probing CCR2-dependent monocyte and hematopoietic recruitment; however, global CCR2 deficiency is not macrophage-specific [15,16]. Previous studies in the ischemic button model demonstrated that macrophage polarization influences adhesion development, with M2-associated markers and arginase activity linked to reduced adhesions, whereas macrophage-specific PPAR-γ deficiency aggravated adhesion formation and pharmacological PPAR-γ activation was protective [2]. Similarly, studies of postoperative ileus showed that recruited monocyte-derived macrophages coordinate both inflammatory and regulatory responses after abdominal surgery through IL-1 and IL-10 signaling pathways [17,18]. Consistent with this concept, a recent study identified CD163^+^ macrophages as an anti-adhesive population. These cells reduced mesothelial PAI-1 secretion and enhanced fibrinolytic activity, thereby limiting postoperative adhesion formation [19]. The present study was designed to examine the contribution of macrophage-rich peritoneal and mesenteric compartments, the hematopoietic origin of macrophage-like cells accumulating in ischemic buttons, and the association between CCR2-dependent recruitment and postoperative adhesion formation. We used clodronate liposomes, bone marrow chimerism, and global CCR2 deficiency as complementary, but not cell-type-exclusive, approaches. Our objective was to determine whether these experimentally defined perturbations were differentially associated with adhesion formation and postoperative gene-expression responses, without presuming that they selectively identify resident or infiltrating macrophage subsets.

## 2. Materials and Methods

### 2.1. Experimental Design

Peritoneal adhesions were induced in mice using the ischemic button model of standardized focal peritoneal injury, a well-established approach for studying adhesion pathogenesis and previously used in mechanistic studies of macrophage polarization and adhesion formation [2]. Experimental groups included vehicle-treated and clodronate liposome-treated mice, wild-type and CCR2-deficient mice, and bone marrow chimeras generated to trace hematopoietic cell origin. Adhesions were scored at postoperative day 7 and quantified by using an adhesion score: score 0, no adhesions; score 1, thin, pellucid adhesions; score 2, tensile adhesion; score 3, inseparable and vascularized adhesion; and score 4, entire abdomen linked by adhesions [2]. Cellular and transcriptional analyses were performed at postoperative day 1 or day 3 as specified. No criteria were set a priori for including and excluding animals during the experiment. No randomisation was used to allocate experimental units. During group allocation, the investigator responsible for assigning participants to the intervention groups was aware of the allocation sequence. Outcome assessment and data analysis were performed by at least one additional assessor who was blinded to group allocation. No formal study protocol was prepared or publicly registered before the commencement of the study.

### 2.2. Animals

Experiments were performed with WT 8- to 12-week-old male C57BL6/J mice (Janvier, Saint Berthevin Cedex, France) with a mean body weight of 20–25 g. Additionally, CCR2^−/−^, CD45.1 were obtained from Jackson Laboratories (Charles River, Sulzfeld, Germany). Animals were housed under specific pathogen-free conditions with controlled temperature and humidity and maintained on a 12 h light/dark cycle with ad libitum access to food and water. All experiments were performed in accordance with federal law for animal protection and approved by the committee for animal experiments of North-Rhine-Westfalia (Approval Code: 84-02.04.2014.A509). Only male mice were used. Therefore, the findings may not generalize to female mice, in which peritoneal immune composition, inflammatory responses, fibrinolytic activity, and adhesion formation may differ.

### 2.3. Bone Marrow Transplantation

Recipient mice received 9 Gy total-body irradiation as a single dose for myeloablative conditioning. Bone marrow cells (BM) were collected from the femur and tibia of CD45.1 donor mice and administered intravenously to recipient mice at a dose of 1.2 × 10^7^ cells per mouse, 7 h after irradiation. No antibiotic prophylaxis was used. The reconstitution period between bone marrow transplantation and ischemic button surgery was 8 weeks. Because irradiation does not necessarily eliminate radioresistant tissue-resident macrophages, donor-marker positivity was interpreted as evidence of donor hematopoietic origin rather than as a complete phenotypic definition of an infiltrating macrophage subset. All experiments were performed in accordance with federal law regarding animal protection and were approved by the state agency for nature, environment, and consumer protection (LANUV).

### 2.4. Ischemic Button Experiments

Potential confounders were minimized by standardizing the surgical procedures and outcome assessments, with all animals undergoing the same ischemic button protocol, the same anesthetic and analgesic regimen, and the same postoperative sampling time points. Housing conditions were also kept uniform under SPF conditions with controlled temperature and humidity, a 12 h light/dark cycle, and ad libitum food and water. Surgery was performed under aseptic conditions. Anaesthesia was induced using isoflurane (Abbott, Wiesbaden, Germany). For analgesia, animals received carprofen 5 mg/kg body weight subcutaneously. Peritoneal adhesion formation was induced by the construction of four buttons on the peritoneal wall. Via a median laparotomy, the peritoneum was lifted with a clamp, and a ligature was applied by first stitching through the base of the button and then ligating the peritoneum. Two buttons were placed on both sides of the peritoneum using a Vicryl^®^ 6/0 suture (Ethicon, Somerville, NJ, USA). The abdomen was closed with a double-layered suture of the peritoneum (Vicryl ^®^ 5/0) and skin (silk 5/0; Braun, Sempach, Switzerland).

### 2.5. Clodronate Liposome Treatment

Macrophage-rich compartments were depleted using 0.2 mL liposomes containing liposome-encapsulated dichloromethylene diphosphonate (CL2MDP) (50 mg clodronat/kg) as shown before. One day before surgery, 0.2 mL CL2MDP liposomes were administered i.p. Vehicle liposomes served as controls. Clodronate liposomes target phagocytic cells and are not exclusively specific for resident macrophages. Local and systemic effects on monocytes, selected dendritic cell populations, and other phagocytic cells cannot be excluded. The intervention was therefore interpreted as depletion of macrophage-rich compartments rather than selective depletion of resident macrophages.

Depletion efficacy was assessed in peritoneal lavage fluid and mesothelial window preparations. The intervention was chosen because transient pharmacologic macrophage depletion has previously been used in abdominal surgery models and can profoundly reduce macrophage abundance during the early postoperative interval [20].

### 2.6. Cytokine and Marker Analysis

Expression of selected inflammatory and macrophage-associated transcripts in ischemic buttons and control peritoneum was assessed at postoperative days 1 and 3. No additional postoperative time points were analyzed. Gene expression of selected inflammatory and wound-healing-associated markers was analysed by PCR. Reagents were from Life Technologies unless specified otherwise. Total RNA was extracted with Trizol^®^ reagent using a tissue homogenizer (Precellys^®^ 24; Peqlab, Erlangen, Germany), followed by DNase I treatment. cDNA was synthesized using a High-Capacity cDNA rt kit (Life Technologies, Carlsbad, CA, USA). Expression of mRNA was quantified in triplicate by reverse transcription-PCR with specific probes/primers (Table S1, Supplementary Materials). The PCR was performed in Power SYBR ^®^ Green or Universal PCR Master Mix by amplification of 10 ng cDNA for 40 cycles (95°C for 15 s, 60°C for 1 min) on an AbiPrism^®^ 7900HT (Life Technologies). Data quantification was performed by the ΔΔCT method and value normalized with respect to glyceraldehyde-3-phosphate dehydrogenase (GAPDH) levels.

### 2.7. Immunofluorescence

Cells from IB were isolated after enzymatic digestion in a solution containing collagenase II (Worthington, Lakewood, NJ, USA), Dispase ^®^ II (La Roche, Mannheim, Germany), DNase (La Roche, Darmstadt, Germany), bovine serum albumin, and trypsin inhibitor. Cells were centrifuged onto glass slides (cytospin method) and stained with rat anti-mouse F4/80 antibody (BM8; Life Technologies, Darmstadt, Germany) and CD 45 antibody (30-F11; ebioscience, San Diego, CA, USA). The nucleus was stained using 4′,6-diamidino-2-phenylindole (DAPI) (Life Technologies, Carlsbad, CA, USA). Cells were counted in five randomly chosen areas in each specimen at a magnification of ×200.

F4/80 staining was not considered sufficient to distinguish large resident peritoneal macrophages, small recruited macrophages, monocytes, neutrophils, or other myeloid populations. In the absence of a multiparameter flow-cytometric panel, F4/80^+^ cells are therefore referred to as F4/80^+^ or macrophage-like cells. CD45.1 positivity was interpreted as evidence of donor hematopoietic origin. Accordingly, CCR2 deficiency was interpreted at the level of CCR2-dependent hematopoietic recruitment rather than as a macrophage-subset-specific perturbation.

### 2.8. Animal Euthanasia

After indicated time points, animals were sacrificed for further analysis. Euthanasia was performed by cervical dislocation.

### 2.9. Statistics

Statistical analysis was performed with Prism V5.04 (GraphPad, San Diego, CA, USA) using one-way ANOVA with multiple comparisons and unpaired *t*-tests and displayed as means + SEM. Data were considered statistically significant at *p*-values < 0.05 (*), <0.01 (**), and <0.001 (***). Continuous data are presented as mean ± SEM unless otherwise stated. For comparisons between two independent groups, unpaired *t*-tests were used when assumptions were met. For comparisons involving more than two groups, one-way analysis of variance was used with the multiple-comparison correction specified in the corresponding figure legend. Prior to statistical analysis, the assumptions underlying each statistical test were evaluated. The distribution of continuous variables was assessed using visual inspection of histograms and Q–Q plots, supplemented by the Shapiro–Wilk test for normality. Homogeneity of variances was evaluated using Levene’s test.

## 3. Results

### 3.1. Depletion of Macrophage-Rich Peritoneal and Mesenteric Compartments Is Associated with Reduced Adhesion Formation

**Figure 1:**
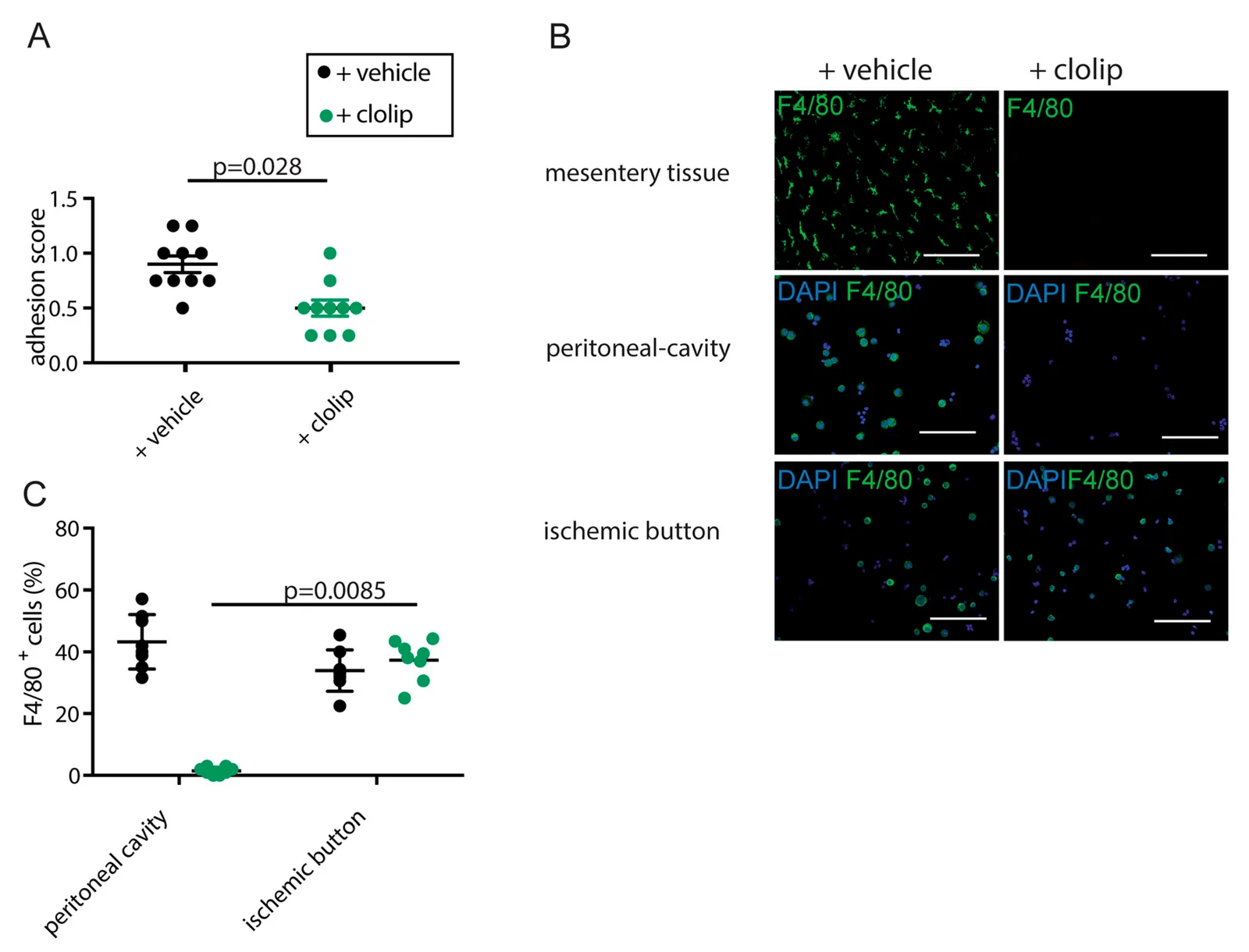
Depletion of macrophage-rich compartments is associated with reduced postoperative adhesion formation. (**A**) Adhesion scores at postoperative day 7 after ischemic button surgery in vehicle-treated and clodronate liposome-treated mice. (**B**) Representative immunofluorescence staining of F4/80^+^ cells and DAPI-labeled nuclei (original magnification ×200; scale bar, 100 μm). (**C**) Quantification of F4/80^+^ cells in peritoneal lavage and ischemic button compartments at postoperative day 3. Values are mean (SEM) (n = 8 vehicle-treated; n = 8 clodronate liposome-treated). Statistical analysis: unpaired Student’s *t*-test for panel (**A**) and one-way ANOVA with Bonferroni post hoc testing for panel (**C**).

Clodronate liposome treatment was associated with a significant reduction in adhesion scores on postoperative day 7 compared with vehicle treatment (*p* = 0.028; Figure 1A). Depletion was substantial in mesenteric tissue and peritoneal lavage, with reductions exceeding 95% and 98%, respectively (Figure 1B,C). However, F4/80^+^ cell numbers within ischemic buttons on postoperative day 3 were not significantly different between clodronate- and vehicle-treated animals.

These findings indicate that depletion of macrophage-rich peritoneal and mesenteric compartments was associated with reduced adhesion formation. They do not establish that all macrophages within the ischemic button were depleted or that resident macrophages were the sole cellular mediators of the observed phenotype. Persistence of F4/80^+^ cells may reflect incomplete drug penetration, early effects of depletion not captured at day 3, recruitment or replacement by other phagocytic cells, or limitations of F4/80-based enumeration.

### 3.2. Postoperative Gene-Expression Responses After Clodronate Treatment

Based on previous findings, the differential expression of IL1β, IL6, Arg1, MR1, and IL10 reflects the dynamic, two-phase inflammatory response that occurs during peritoneal wound healing and adhesion formation after abdominal surgery [2]. Therefore, proinflammatory markers were measured at postoperative day 1, while tissue repair markers were measured at postoperative day 3.

**Figure 2:**
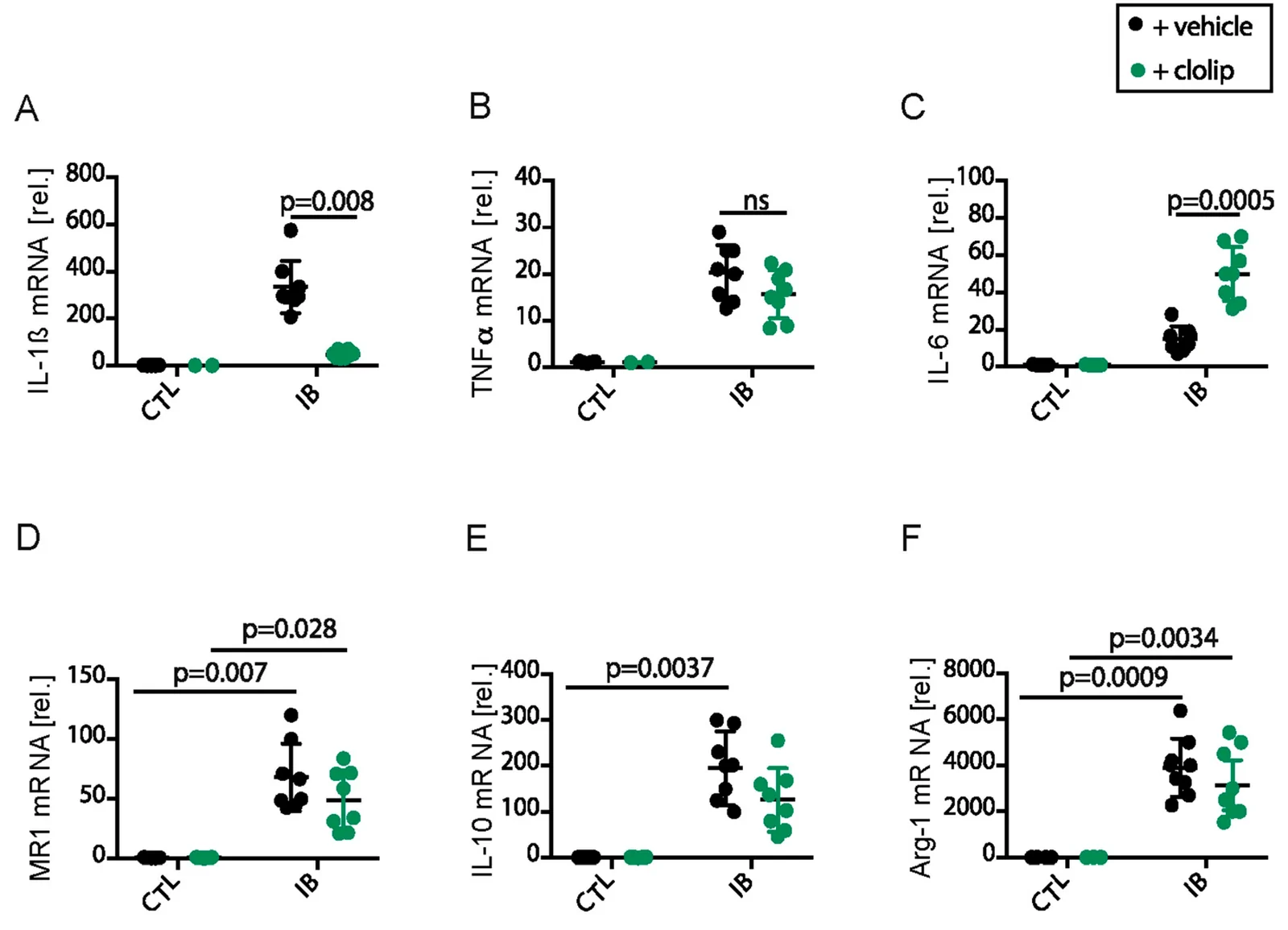
Postoperative inflammatory and wound-healing-associated transcript expression after clodronate treatment. Quantitative PCR analysis of IL1β (**A**), TNF-α (**B**), and IL6 (**C**) at postoperative day 1 and Arg1 (**D**), MR1 (**E**), and IL10 (**F**) at postoperative day 3 in control peritoneum and ischemic button tissue from vehicle-treated and clodronate-treated mice. Values are mean (SEM) (n = 8 vehicle-treated; n = 8 clodronate liposome-treated). Statistical analysis: one-way ANOVA with Bonferroni post hoc testing.

At postoperative day 1, IL1β and IL6 expression were markedly increased in ischemic buttons compared with control peritoneum (Figure 2A,C). IL1β was significantly elevated in vehicle-treated ischemic buttons versus vehicle control peritoneum (*p* = 0.0004), while ischemic buttons from clodronate-treated mice also differed from vehicle-treated ischemic buttons (*p* = 0.008), showing that IL1β transcript expression was altered by clodronate treatment. Given the non-specificity of clodronate, this finding is compatible with a contribution of clodronate-sensitive phagocytic cells but does not identify a resident macrophage-specific source. IL1B, TNF, and IL6 results are presented as transcript expression. These findings are compatible with a contribution of macrophage-rich compartments to selected inflammatory responses but do not establish a macrophage-exclusive mechanism.

TNF-α did not show a significant difference in the reported comparison between clodronate- and vehicle-treated ischemic buttons (Figure 2B), whereas IL6 was strongly induced in injured lesions and differed between ischemic buttons from clodronate- and vehicle-treated animals (*p* = 0.0005). Because IL6 can also be induced by tissue injury and by non-macrophage stromal or immune cells, this pattern is consistent with a mixed cellular origin of early cytokine production and does not support a macrophage-exclusive mechanism.

Arg1, MR1, and IL10 (day 3) were all significantly increased in ischemic buttons relative to control peritoneum (MR1: *p* = 0.007 (+vehicle), *p* = 0.028 (+clolip); IL10: *p* = 0.0073 (+vehicle); Arg1: *p* = 0.0009 (+vehicle), *p* = 0.0034 (+clolip)) (Figure 2D–F). Although some of these markers trended lower after clodronate treatment without reaching significance in all pairwise comparisons, the overall pattern shows early induction of wound-healing-associated transcripts in injured tissue. No consistent significant effect of clodronate treatment was observed across all three wound-healing-associated transcripts. These data document wound-healing-associated transcriptional responses but do not establish a specific M2 macrophage population, the cellular source of the transcripts, or protein-level cytokine activity.

### 3.3. Donor-Derived Hematopoietic Cells Are Present in Ischemic Buttons

**Figure 3:**
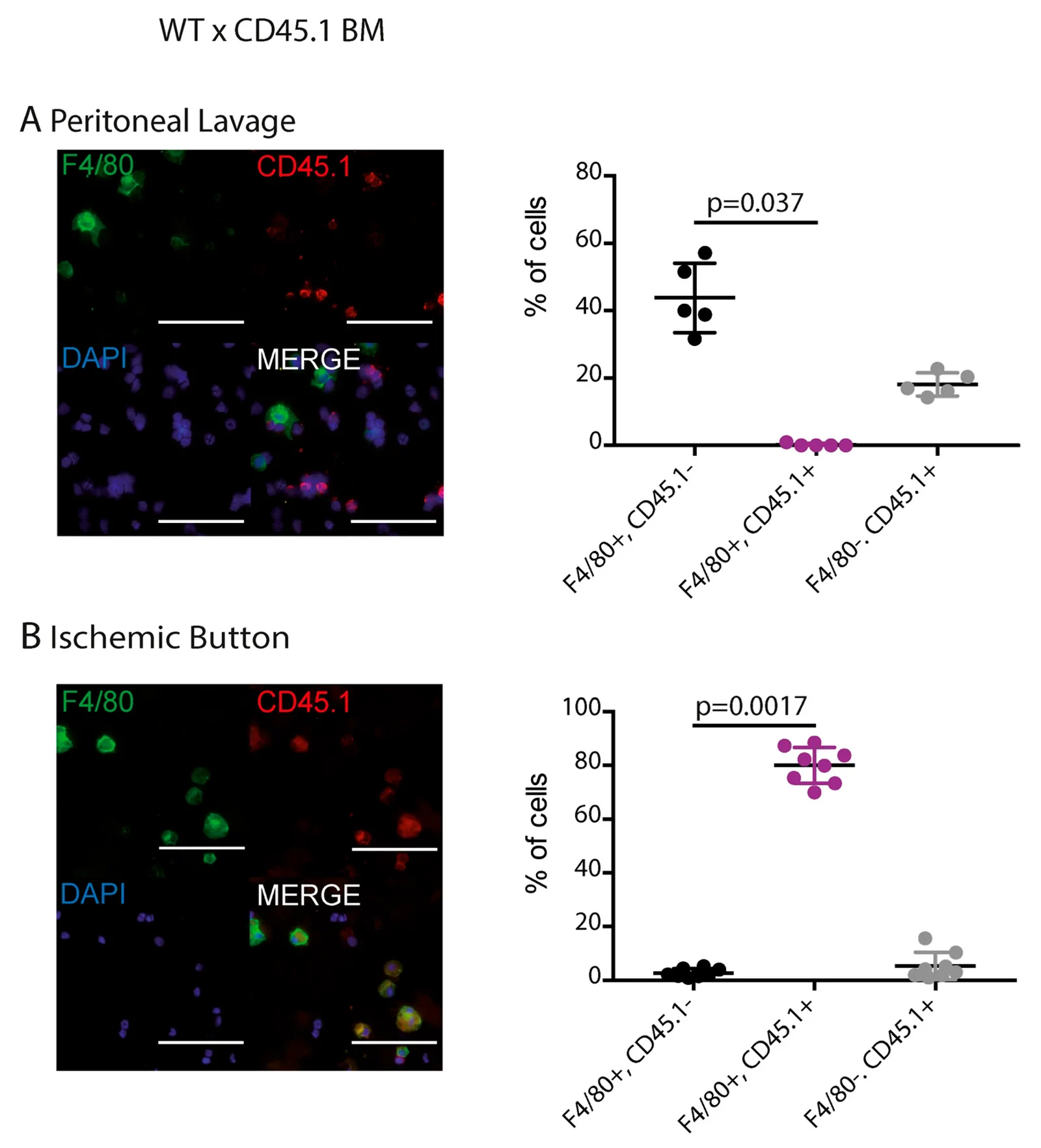
Donor-derived hematopoietic cells are present in ischemic buttons after bone marrow transplantation. Representative immunofluorescence staining and quantification of F4/80^+^CD45.1^+^, F4/80^+^CD45.1^−^, and F4/80^−^CD45.1^+^ cells in (**A**) peritoneal lavage and (**B**) ischemic button samples from bone marrow chimeric mice at postoperative day 3. Nuclei were stained with DAPI. Original magnification ×200; scale bar, 100 μm. CD45.1 positivity is interpreted as evidence of donor hematopoietic origin; F4/80 positivity alone does not establish a macrophage-subset identity.

To assess whether donor-derived hematopoietic cells contribute to the lesional F4/80^+^ population, bone marrow chimera experiments were performed. Shifts in F4/80^+^ CD45.1^+^ cell populations in both lavage and ischemic button samples supported recruitment of hematopoietic donor-derived cells into the lesion (Figure 3). In lavage samples, donor-marker-positive populations were significantly represented in several pairwise comparisons, and in ischemic buttons, the comparison between F4/80^+^ CD45.1^−^ and F4/80^+^ CD45.1^+^ populations favored donor-marker-positive F4/80^+^ lesional cells (Figure 3A,B; *p* < 0.001).

These data support recruitment of donor-derived hematopoietic cells with a macrophage-like phenotype into the lesion. Because the analysis was based on F4/80 and CD45.1 rather than multiparameter flow cytometry or fate mapping, the data do not fully resolve resident macrophages from recruited monocytes, monocyte-derived macrophages, or other myeloid populations.

### 3.4. CCR2 Deficiency Alters Lesional Cell Accumulation and Adhesion Severity

**Figure 4:**
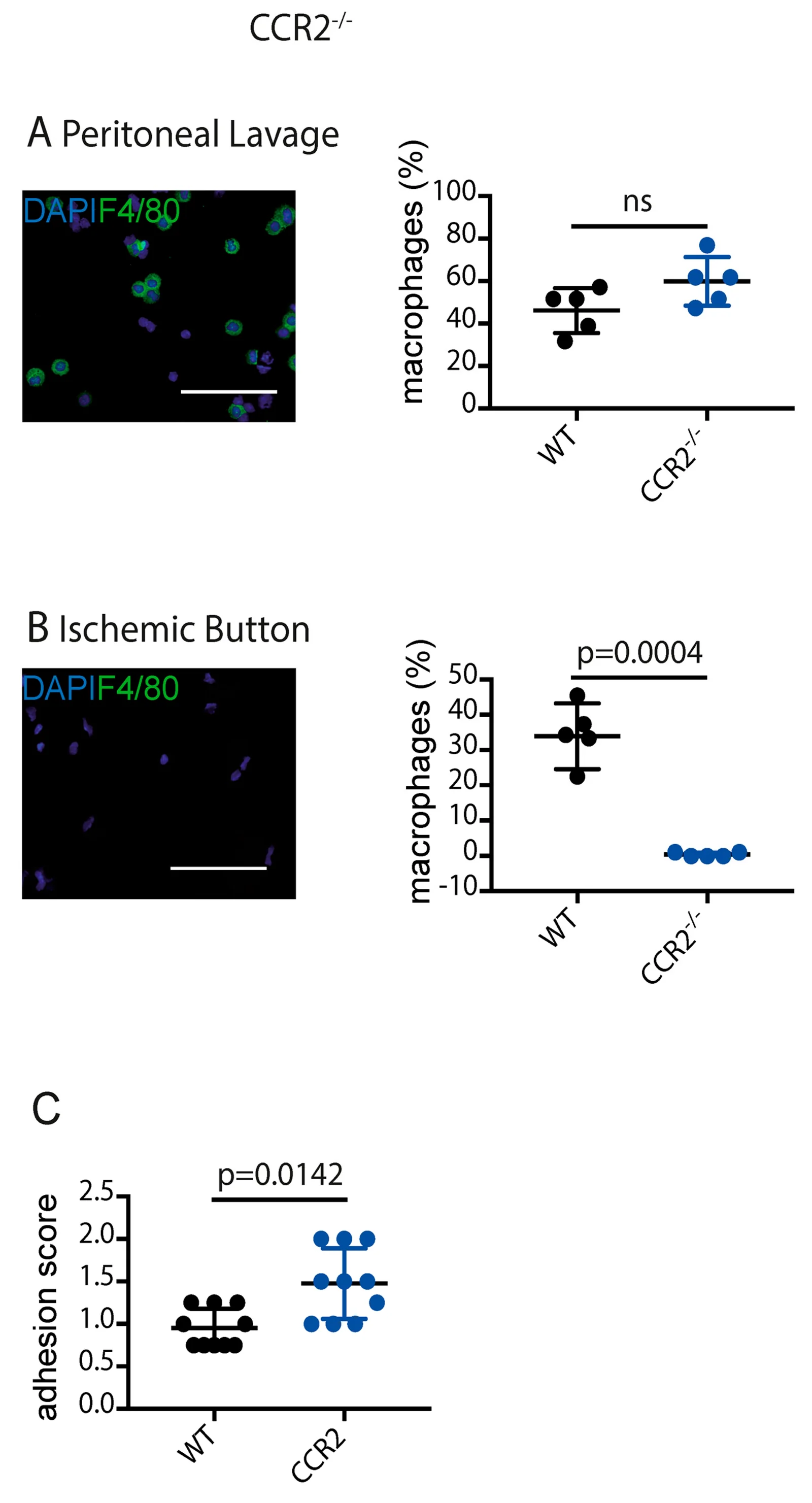
Global CCR2 deficiency alters lesional F4/80^+^ cell accumulation and adhesion severity. Quantification of F4/80^+^ cells in (**A**) peritoneal lavage and (**B**) ischemic button tissue at postoperative day 3 and (**C**) adhesion scores in wild-type and CCR2-deficient mice. Values are mean (SEM) (n = 10 WT; n = 10 CCR2^−/−^). Statistical analysis: unpaired Student’s *t*-test for pairwise comparisons.

CCR2 deficiency did not significantly alter peritoneal F4/80^+^ cell abundance (Figure 4A) but significantly reduced F4/80^+^ cell accumulation within ischemic buttons at postoperative day 3 (*p* = 0.0004; Figure 4B). Adhesion scores were also significantly higher in CCR2-deficient mice than in wild-type controls (*p* = 0.0142; Figure 4C). CCR2 deficiency reduced F4/80^+^ cell accumulation within ischemic buttons and was associated with increased adhesion scores. These findings associate intact CCR2 signaling/recruitment with greater lesional F4/80^+^ cell accumulation and lower adhesion severity in this model. Because CCR2 is not restricted to macrophages or monocytes and the animals carried a global CCR2 deficiency, the phenotype cannot be attributed specifically to macrophages.

### 3.5. CCR2 Deficiency Is Associated with Altered Inflammatory and Wound-Healing-Associated Transcripts

**Figure 5:**
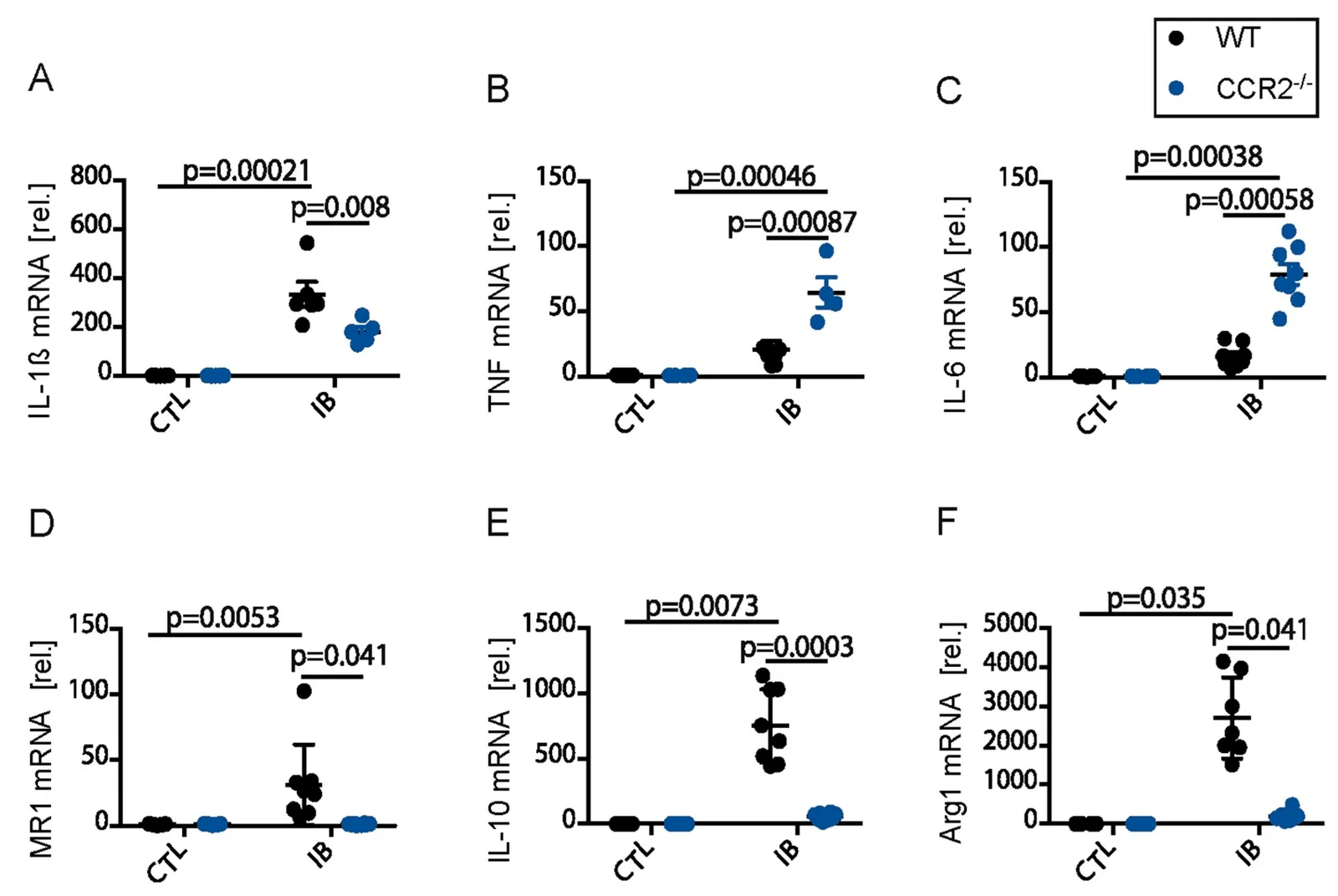
Global CCR2 deficiency is associated with altered inflammatory and wound-healing-associated transcript expression. Quantitative PCR analysis of IL1β (**A**), TNF-α (**B**), and IL6 (**C**) at postoperative day 1 and MR1 (**D**), IL10 (**E**), and Arg1 (**F**) at postoperative day 3 in control peritoneum and ischemic button tissue from wild-type and CCR2-deficient mice. Values are mean (SEM) (n = 8 WT; n = 8 CCR2^−/−^). Statistical analysis: one-way ANOVA with Bonferroni post hoc testing.

To examine the association between CCR2-dependent recruitment and postoperative inflammatory transcriptional responses, mice lacking the chemokine receptor CCR2, which is required for efficient mobilization and recruitment of inflammatory monocytes, were examined following ischemic button surgery. At postoperative day 1, TNF-α was significantly increased in ischemic buttons compared with control tissue in CCR2-deficient mice (*p* = 0.00046), and TNF-α levels were also significantly higher in ischemic buttons from CCR2-deficient mice than in wild-type ischemic buttons (Figure 5B; *p* = 0.00087), indicating that the early TNF-α response persists despite CCR2 deficiency. IL6 showed the same overall pattern, with strong induction in ischemic buttons and a significant difference between CCR2-deficient and wild-type ischemic buttons (Figure 5C; *p* < 0.00058), showing that early inflammatory signaling persisted in the setting of impaired CCR2-dependent recruitment. IL1β was also robustly induced after surgery and significantly elevated in ischemic buttons versus control tissue in both CCR2-deficient (*p* = 0.00037) and wild-type mice (*p* = 0.00021). However, IL1β levels were lower in ischemic buttons from CCR2-deficient mice than in wild-type ischemic buttons (Figure 5A; *p* = 0.008).

At postoperative day 3, wound-healing-associated transcripts were also induced. MR1 was significantly increased in ischemic buttons compared with control tissue (*p* = 0.0053), whereas MR1 expression in CCR2-deficient ischemic buttons was significantly decreased relative to wild-type ischemic buttons (Figure 5D; *p* = 0.041). IL-10 showed a similar pattern, with significantly higher expression in ischemic buttons than in control tissue (*p* = 0.0073) and a significant difference between wild-type and CCR2-deficient ischemic buttons (Figure 5E; *p* = 0.0003). Arg-1 was likewise increased in ischemic buttons versus controls (*p* = 0.035) and differed significantly between wild-type and CCR2-deficient ischemic buttons (Figure 5F; *p* =0.041), indicating that global CCR2 deficiency was associated with lower day 3 expression of MR1, IL10, and Arg1 in ischemic buttons. Because expression was measured in whole tissue and CCR2 deficiency was not cell-specific, these data do not establish that CCR2-dependent recruited macrophages produced these transcripts or acquired a reparative, anti-inflammatory, or pro-resolving phenotype.

## 4. Discussion

Postoperative peritoneal adhesions remain a major clinical problem because they are frequent, difficult to prevent, and associated with chronic pain, bowel obstruction, infertility, and reoperation risk [19,21]. This study identifies different associations between clodronate-sensitive, macrophage-rich peritoneal and mesenteric compartments and CCR2-dependent hematopoietic recruitment. These experimentally defined perturbations show different relationships with adhesion development but do not resolve phenotypically defined resident and infiltrating macrophage subsets. The CCR2-deficient phenotype is compatible with an adhesion-limiting association of intact CCR2-dependent recruitment in this model. However, global CCR2 deficiency affects monocyte mobilization and potentially other CCR2-expressing leukocyte populations. The observed phenotype should therefore be interpreted as the consequence of altered CCR2-dependent hematopoietic recruitment rather than macrophage-specific evidence. This is in line with a previous study showing that M2-like macrophage polarization and macrophage PPAR-γ signaling protect against adhesion formation [2]. Accordingly, the present data should be viewed as hypothesis-generating evidence for potentially divergent contributions of macrophage-rich compartments and recruited hematopoietic cells, rather than proof that resident and infiltrating macrophage populations have distinct and opposing functions in adhesion pathogenesis.

A key finding of our work is that clodronate-mediated depletion of macrophage-rich peritoneal and mesenteric compartments was associated with reduced adhesion formation. The reduction in adhesion formation after clodronate treatment indicates that depletion of one or more clodronate-sensitive phagocytic populations alters the adhesion outcome. It does not identify which phagocytic population mediates this effect. Nevertheless, the intervention was not selective for resident macrophages. Moreover, F4/80^+^ cell numbers in ischemic buttons were not reduced at day 3. Thus, the present data cannot determine whether the adhesion phenotype resulted from depletion of resident cells before lesion formation, altered early signaling, effects on other phagocytic populations, or a combination of these mechanisms. This is consistent with the concept that peritoneal macrophages are among the first responders to sterile injury and participate in fibrin containment, cytokine release, and mesothelial remodeling, processes that can either favor repair or, when dysregulated, drive pathological adhesion formation. Human and experimental studies have likewise shown that macrophages are present early after adhesion induction and that persistent inflammatory activity can remain detectable even in mature adhesions [22,23,24].

At the same time, persistence of lesional F4/80^+^ cells at postoperative day 3 despite efficient depletion in lavage and mesenteric compartments argues against equating the clodronate-sensitive compartments with the entire lesional myeloid population. This discrepancy could reflect incomplete drug penetration, temporal repopulation, recruitment of circulating cells, or effects on other phagocytic populations. The present data do not establish which mechanism predominates, nor do they establish the tissue source or ontogeny of the persistent lesional F4/80^+^ cells.

The presence of CD45.1^+^F4/80^+^ cells in ischemic buttons supports recruitment of donor-derived hematopoietic cells with a macrophage-like phenotype. However, radioresistant resident macrophages may persist after irradiation, and F4/80/CD45.1 staining does not provide the phenotypic resolution required to identify all resident and recruited subsets. This finding is in line with broader literature showing that monocyte recruitment to inflamed tissues is a key determinant of postoperative immune responses and that tissue macrophage ontogeny strongly shapes effector function [8,25,26].

CCR2 deficiency provided complementary evidence at the level of CCR2-dependent recruitment. CCR2-deficient mice were compared with wild-type controls to examine the relationship between CCR2-dependent recruitment, lesional F4/80+ cell accumulation, and adhesion formation. This approach was guided by prior postoperative ileus studies showing that CCR2 is central to monocyte trafficking into surgically traumatized bowel wall and thus may represent a shared recruitment pathway across postoperative inflammatory syndromes [17,18]. We found that loss of CCR2 reduced F4/80^+^ cell accumulation within ischemic buttons but increased adhesion severity. This shows that impaired CCR2-dependent recruitment coincided with greater adhesion severity and argues against a simple model in which CCR2-dependent recruitment is uniformly adhesion-promoting during adhesion formation; however, the present experiment does not identify the responsible CCR2-expressing cell type or mechanism. Similar protective roles for CCR2-dependent monocyte-derived macrophages have been described in postoperative ileus, where recruited cells restore tissue homeostasis and promote recovery rather than amplify injury [16]. Together with the chimera data, these findings support donor-derived hematopoietic recruitment into the lesion and associate CCR2-dependent recruitment with adhesion severity, without defining the function of a specific macrophage subset. More recently, a dedicated adhesion study also showed that monocyte-derived peritoneal macrophages can protect mice against surgery-induced adhesions and that CCR2 deficiency or depletion of CCR2^+^ monocytes worsens adhesion formation [8,16]. Prior literature raises the hypothesis that monocyte-derived macrophages may restrain excessive fibrin persistence, coordinate clearance of injury-associated debris, and contribute to resolution; these functions were not directly demonstrated by the present experiments.

However, the study assessed selected postoperative time points rather than a complete time course. Therefore, the data do not directly demonstrate temporally separated resident and infiltrating macrophage functions. The reduction in adhesion after depletion of macrophage-rich peritoneal and mesenteric compartments is compatible with an early effect of clodronate-sensitive cells, while the day-3 chimera and CCR2 findings indicate that donor-derived hematopoietic cells are present during the postoperative response. Definitive temporal relationships will require serial analyses at additional time points, such as postoperative days 1, 3, 5, and 7, using markers that resolve resident, monocyte-derived, and other myeloid populations. The present experiments also do not link donor-derived F4/80^+^ cells to the CCR2-dependent phenotype at a single-cell level.

Our cytokine data support this interpretation by showing that the early inflammatory response after surgery is only partially dependent on CCR2. IL1β was strongly induced in ischemic buttons and was partially sensitive to macrophage depletion, consistent with established work in postoperative ileus showing that IL-1 signaling is a central driver of postoperative neuroimmune activation [18]. Because enteric glia respond to IL-1β with production of IL-6 and monocyte-attracting signals in postoperative ileus models, the present lesion data raise the possibility that analogous stromal or mesothelial amplification loops may operate in the peritoneum, even though this was not directly tested here [18]. However, the study relied primarily on mRNA expression and did not include ELISA, Western blot, or other protein-level validation. Therefore, the observed changes in IL1B, TNF, IL6, ARG1, MRC1, and IL10 transcripts cannot establish corresponding changes in protein abundance, secretion, or functional activity.

The results also fit with the broader concept that recruited monocyte-derived macrophages can generate regulatory mediators while still worsening postoperative pathology. Stein et al. showed that leukocyte-derived IL-10, chiefly from monocyte-derived macrophages, aggravates postoperative ileus by promoting chemokine expression and neutrophil extravasation rather than by suppressing inflammation in a straightforward manner [17]. Of particular interest is the pattern observed for Arg1, MR1, and IL10, which were increased in injured tissue at day 3 and were reduced in CCR2-deficient lesions. In the present study, this pattern demonstrates an association between intact CCR2 signaling/recruitment and higher whole-tissue expression of wound-healing-associated transcripts at day 3. It does not establish the cellular source of these transcripts, their protein-level activity, or a reparative, anti-inflammatory or pro-resolving phenotype of CCR2-dependent recruited macrophages. Such a phenotype remains a plausible hypothesis for future cell-specific investigation [22,27].

These findings also help reconcile apparently conflicting literature on macrophages in adhesion formation. Some studies have emphasized macrophage-driven profibrotic signaling and the ability of macrophages to induce fibroblast adhesion phenotypes, whereas others have shown that monocyte-derived macrophages can be protective depending on timing, localization, and polarization state [22,26,28]. Rather than supporting a definitive temporal and functional dichotomy, the present work identifies two divergent experimental phenotypes: clodronate-sensitive, macrophage-rich compartment depletion reduced adhesions, whereas global CCR2 deficiency increased adhesions and altered lesional F4/80^+^ cell accumulation and transcript expression. Whether these phenotypes reflect distinct resident and infiltrating macrophage functions requires cell-specific lineage tracing and functional validation.

Several limitations should be considered. First, clodronate liposomes are not selective for resident macrophages and may affect other phagocytic cells. Second, F4/80 staining alone does not distinguish resident macrophages, recruited monocytes, monocyte-derived macrophages, and other myeloid populations. Third, the chimera experiments require confirmation of reconstitution and cannot exclude persistence of radioresistant resident cells. Fourth, the CCR2-deficient model is global and therefore does not establish macrophage-specific causality. Fifth, the study relies primarily on mRNA expression without protein-level validation. Sixth, lesional F4/80^+^ cell content was assessed at limited postoperative time points, preventing a definitive temporal analysis. Seventh, only male mice were studied, limiting generalizability across sex. Finally, the ischemic button model is valuable for mechanistic investigation but does not reproduce all aspects of human postoperative adhesion disease. In addition, whole-tissue qPCR cannot assign transcript changes to recruited F4/80^+^ cells, and expression of Arg1, MRC1/MR1, or IL10 alone is not proof of a pro-resolving cellular function.

## 5. Conclusions

The present findings identify divergent associations between two non-cell-specific perturbations during postoperative adhesion formation. Clodronate-mediated depletion of macrophage-rich peritoneal and mesenteric compartments was associated with reduced adhesion formation, whereas global CCR2 deficiency was associated with reduced lesional F4/80+ cell accumulation, altered inflammatory and wound-healing-associated transcripts, and increased adhesion severity. Because clodronate treatment, F4/80/CD45.1 labeling, bone marrow chimerism, and global CCR2 deficiency do not definitively distinguish resident from recruited macrophage subsets or assign cell-specific function, these results should not be interpreted as proof of opposing resident/infiltrating macrophage roles or of a reparative phenotype in CCR2-dependent cells. Future studies using lineage-resolved, macrophage-specific, and temporally controlled approaches are required before subset-specific therapeutic strategies can be proposed.

## Data Availability

The original contributions presented in this study are included in the article/Supplementary Materials. Further inquiries can be directed to the corresponding author.

## Supplementary Materials

The following supporting information can be downloaded at: https://www.mdpi.com/article/10.3390/biomedicines14092014/s1, Table S1. Primer sets used for gene expression analyses by quantitative PCR.
