# Supplementary material for: Macrophage-Rich Peritoneal Compartments and CCR2-Dependent Hematopoietic Recruitment Are Differentially Associated with Adhesion Formation After Abdominal Surgery
Source: Biomedicines. 2026 Sep 8;14(9):2014. doi: 10.3390/biomedicines14092014 (PMC13604862; doi:10.3390/biomedicines14092014)
Supplement: Supplementary file 1 [file biomedicines-14-02014-s001.zip › biomedicines-4436083-supplementary.pdf]

Table S1 Primer sets used for gene expression analyses by quantitative PCR

	Forward primer<br>5´→ 3´	Reverse primer<br>5´→ 3´	Assay ID
TNF-α			Mm00443258*
IL-1β			Mm00434228_m1*
IL-6	AAGTCGGAGGCTTAATTACACATGT	CCATTGCACAACTCTTTTCTCATT	
Arg1			QT00134288
MR-1			QT00103012*
IL-10	GATGCCCCAGGCAGAGAA	CACCCAGGGAATTCAAATGC	
GAPDH			4352339e*

*Tagman assay (Life Technologies, Darmstadt, Germany), †QuantiTect Primer Assay (Qiagen, Hilden, Germany).
